# A new cleaning method for solar panels inspired from the natural vibrations of tree branches and leaves

**DOI:** 10.1038/s41598-024-68215-y

**Published:** 2024-08-05

**Authors:** M. S. Abd-Elhady, Adil Rana, M. A. Elsebaaie, H. A. Kandil

**Affiliations:** 1 Mechanical Engineering Department, Faculty of Engineering, Beni-Suef University, Beni-Suef, Egypt; 2 Mechatronics Department, Faculty of Engineering and Materials Science, German University in Cairo (GUC), Cairo, Egypt; 3 Department of Electrical Engineering, University of Kassel, Kassel, Germany

**Keywords:** Dust accumulation, Photovoltaic, PV panels, Soiling, Energy science and technology, Engineering

## Abstract

Photovoltaic (PV) panels are similar in many aspects to the leaves of trees, both are standing in the Sun to capture the sunlight, however, PV panels get soiled especially in desert areas, while the leaves remain clean to a very good extent. The question is, *why leaves remain clean while PV panels get soiled quite easily*? The leaves are hanging on the stem of trees and these stems are flexible to motion, such that if the wind blows in any direction over the stem it vibrates allowing any deposited particle to fall off the surface. The objective of this research is to develop a fixation method for PV panels similar to the stems of trees, such that the panel can vibrate as the wind blows in order to minimize dust accumulation. Different fixation methods for the PV panel are designed, and the air flow around the panel is simulated using the CFD package Ansys Fluent. It has been found that a PV panel pivoted at its lower edge, such that it can revolve around the lower edge, together with a vertical wind shield attached to its upper edge and a spring attached at the middle of its backside has the largest vibration amplitude due to the applied wind compared to the other designs. Experiments have been done to infer the influence of the new fixation method of the PV panel on dust accumulation over the panel. After 6 weeks of operation, it has been found that the efficiency of the PV panel that is flexibly fixed has dropped by only 5%, while the efficiency of the panel that is rigidly fixed has dropped by 25%. It can be concluded that a PV panel operating a light post should be fixed on a flexible base that allows the panel to vibrate as the wind blows over it in order to mitigate dust.

## Introduction

### Influence of dust on the performance of PV panels

Many Sunbelt countries are relying nowadays on generating electricity from renewable energy, especially solar energy, due to the rise in fuel prices, global warming and depletion of fossil fuels^1^. The region of the Middle East and North Africa (MENA) is one of the most promising regions in the world for generating electricity from solar energy, due to the long daily duration of sunshine hours^1^. The simplest system for direct conversion of solar energy into electrical energy is Photovoltaic (PV) panels^2^. However, dust accumulation on PV panels represents a major challenge for the operation of panels especially in the MENA region^3^ due to the high rate of dust^4^, scarcity of water that can be used in cleaning^5,6^ in addition to the low frequency and intensity of rainfalls^7^. Dust mitigation of solar photovoltaics is a main aspect of maintenance required for enhanced and longer yield performance of PV panels^8^. Dust and dirt accumulation on the panel's surface impairs the performance of the PV panel as it decreases the output power and consequently lowers the efficiency of the PV panel resulting in decreasing the performance^9^. There is a huge range of researches^10–12^ carried out on the influence of dust on the PV panels performance, and the rate of dust accumulation is a function of the geographical location.

For example, Al-Otaibi et al.^13^ investigated the influence of dust on the performance of a small scale PV system installed on the rooftop of a school in Kuwait, and found that soiling losses amounted for 45.8% over a period of three months without cleaning. Figgis et al.^14^ studied the impact of dust on the energy yield of PV panels installed in Qatar, and it was found that the energy yield has decreased by 15% after 1 month, moreover, the soiling loss, can reach up to 68% without rain or cleaning after 234 days^14^. An experimental study was performed on the effect of dust deposition on the efficiency of photovoltaic panels installed in Baghdad, Iraq^3^, and it was found that the drop in efficiency is equal to 6.24% daily, 11.8% weekly, and 18.74% monthly. Another study was conducted by Al-Hasan and Ghoneim^15^ in Saudi Arabia, and it was recorded that the drop in efficiency due to dust accumulation has reached 33.5% after one month of operation and 65.8% after six months. Several studies^16,17^ have been done to discern the influence of dust on the efficiency of PV panels in Egypt, and it was found that the drop in efficiency could reach 50% in a very short time, i.e. less than 3 months. It can be clearly concluded that dust will be a continuous challenge for solar PV panels, particularly in desert areas, and the PV plant owners in such areas are always in search of cheaper and alternative ways to meet their cleaning needs^18^.

### Dust mitigation techniques

There are many studies that have been implemented to mitigate dust on ground mounted PV panels, however, very little research has been done to mitigate dust on PV panels operating light posts. Cleaning techniques of solar panel can be broadly classified into (1) active techniques, (2) passive techniques and (3) a combination of both techniques. An active technique is an active restoration cleaning method, which utilizes external energy. Mechanical cleaning such as autonomous robots^19–21^ could be a solution for cleaning of PV systems because it has several advantages such as quick response and high reliability in addition to solving the problems of labor. Nevertheless, the initial cost is high in case of robotic cleaning due to the use of various hardware. However, the technique is not feasible for cleaning a single panel installed at elevated heights, because the benefit from cleaning is less than the price of the robot. The Electrodynamic Screen (EDS) cleaning technique^22^ is characterized by consuming very low amounts of energy and it is capable of achieving very high cleaning efficiencies i.e. greater than 90% such that the surface of the PV panel is almost restored to its basic clean conditions^23^. EDS is unreliable in wet conditions or with cemented dust, however, it can be integrated with super hydrophobic coatings in order to achieve a better cleaning effect on the panels in rainy conditions^24,25^. On the other hand, a passive cleaning technique does not make use of external energy. An example for passive cleaning is natural cleaning, which makes use of rainfalls to remove the dust accumulated on the panel’s surface. However, due to the shortage of rainfalls in many regions, especially the MENA region, other passive techniques arise, which are using super hydrophilic or super hydrophobic coatings. A hydrophobic surface is a surface having low wettability. This property helps to repel water droplets and prevents them from sticking on the surface. And, as the water droplets are repelled by the coating and fall down the panel, they carry away the dust from the panels surface.

**Figure 1:**
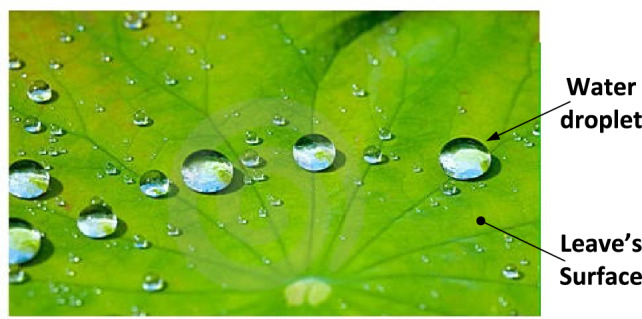
An eminent example of a hydrophobic surface, i.e. leaves, which repels water droplets causing cleaning of the leaf as the droplet rolls down.

An eminent example of a hydrophobic surface is the surface of leaves as can be seen in Fig. 1, such that if there is a water droplet on the leaf it is repelled, and carries away dust as it falls down the surface. The self-cleaning property of leaves was first studied by Dettre and Johnson in 1964^26^, and then the study continued by Wilhelm Barthlott and Ehler in 1977^27^, who described such self-cleaning and ultrahydrophobic properties for the first time as the "lotus effect". Afterwards, many hydrophobic materials were developed for handling chemical and biological fluids^28^, and other biotechnical applications have emerged since the 1990s^29–31^.

**Figure 2:**
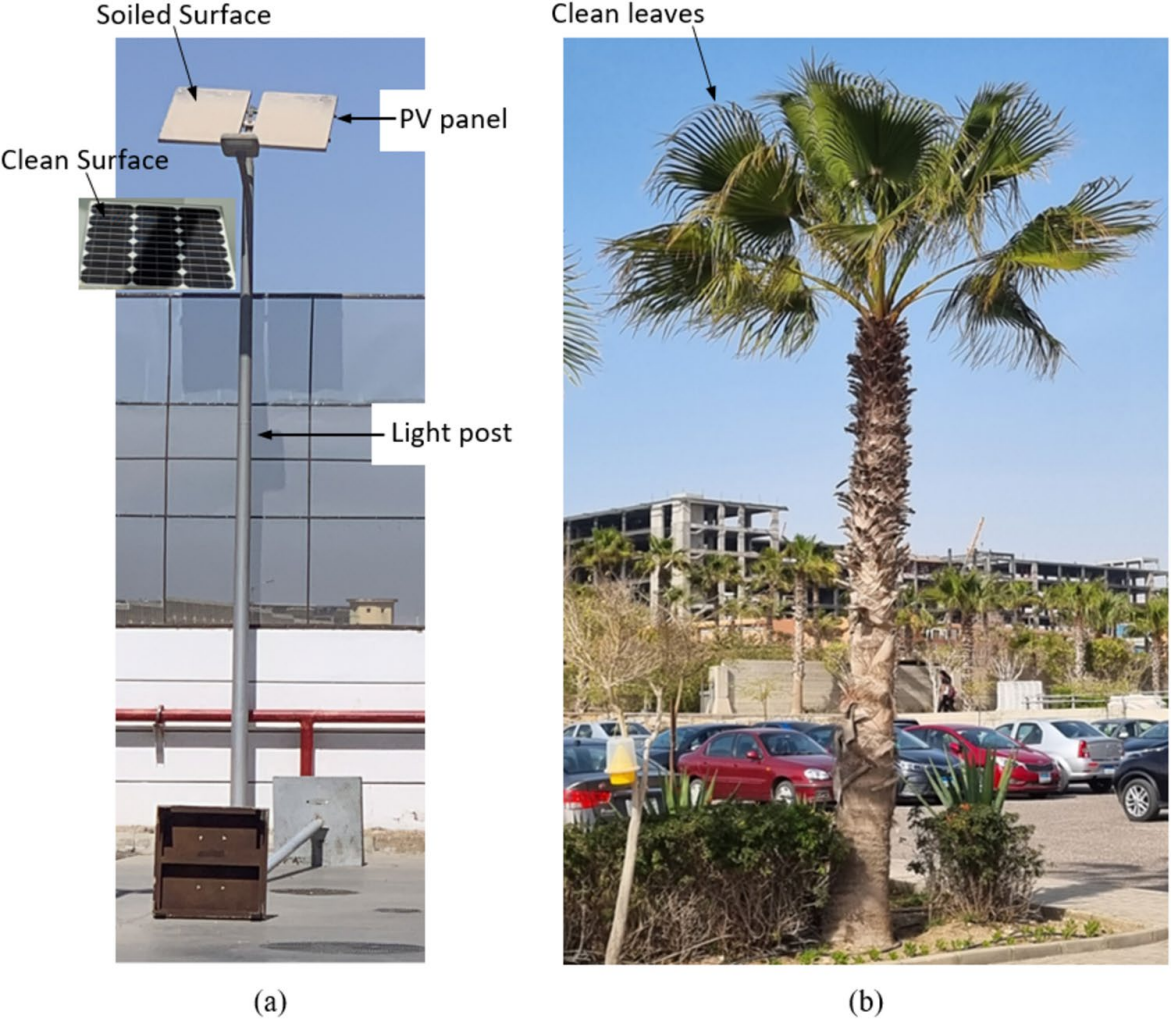
(**a**) A photo of a PV panel operating a light post, in Cairo, Egypt, 2022, which has not been cleaned for three months while (**b**) is a photo of a palm tree that has never been manually cleaned, but it is naturally cleaned.

A hydrophilic surface is a surface having high wettability. Both hydrophilic and hydrophobic surfaces obtain their cleaning effect through rainfalls. Another type of PV panel coating used in dust mitigation is the antistatic coating. This coating prevents the sticking of dust particles to the coated surface such that the particles are removed by the effect of rainfalls. Hence, the benefit of using hydrophilic or hydrophobic coatings depends upon the frequency of raining. The dust on the coated PV panels can be washed out in case of rains which is quite rare in the MENA region. The problem of the dust being on the surface and not wiped off, especially in the MENA region due to the scarcity of rains, destroys the effectiveness of any coating. Al-Badra et al.^32^ developed an automated mechanical vibrator for PV panels coated with an antistatic-hydrophilic coating to overcome the problem of rain scarcity. The function of the vibrator is to shake the panel twice a day, such that the dust on the panel can fall off the surface by gravity. It has been concluded that dust mitigation using coatings is an effective technique in cleaning solar panels, and such a performance can be improved if a vibration system is applied. Shenouda et al.^33^ investigated the influence of the vibration time and amplitude on the accumulation of dust over the PV panel, and it is found that increasing any of the previous parameter results in decreasing soiling of the panel. Cleaning of the PV panels is not an easy task and is expensive especially when the panels are installed at elevated heights and in the desert, i.e. a dry climate. A photo of a PV panel that has not been cleaned for 3 months, and a photo of a palm tree that has never been cleaned manually, in Cairo, Egypt, are shown in Fig. 2. Both leaves and PV panels are standing in the sun to capture the sun light, however, PV panels get soiled easily, while the leaves remain clean to some extent that keeps it alive even when it is subjected to strong sand storms. The question is why leaves don’t get soiled, while PV panels are? To answer this question it is very important to monitor the behavior of trees/leaves in desert areas and by learning how leaves remain clean, it might be possible to develop a similar cleaning technique for PV panels.

**Figure 3:**
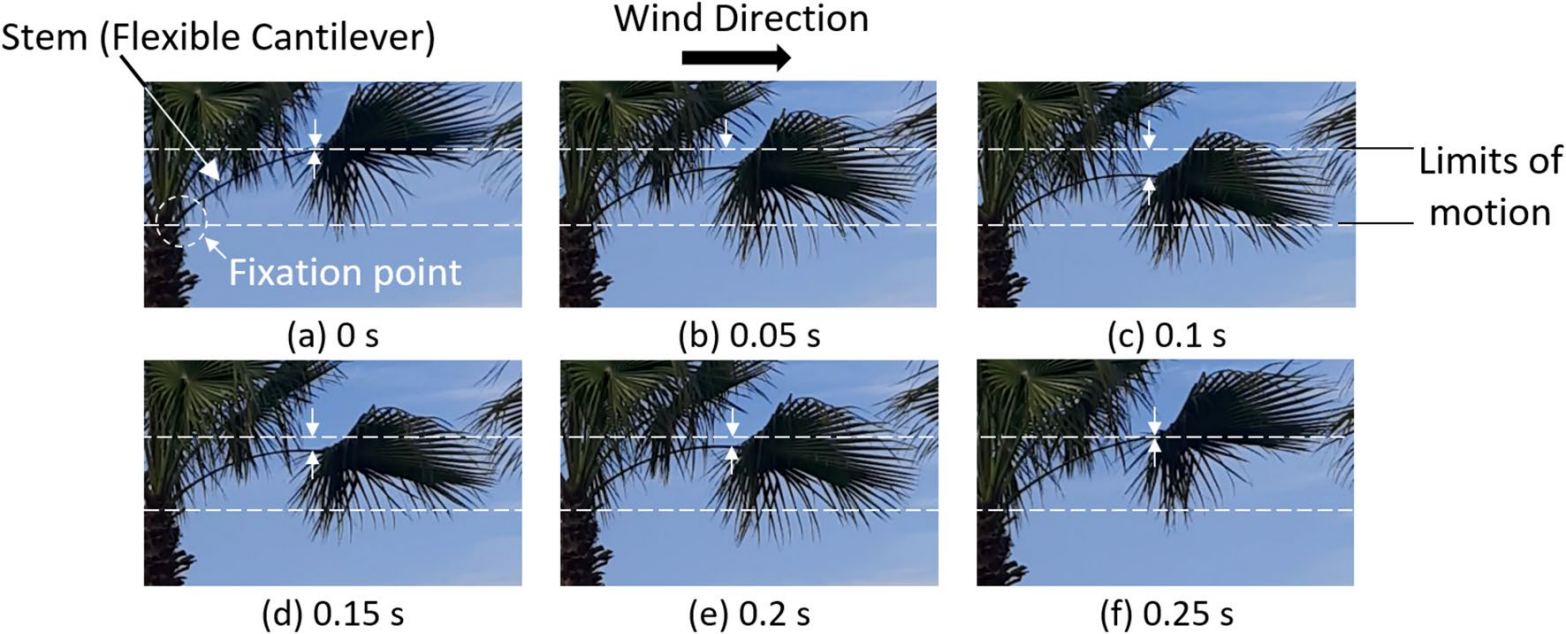
Oscillations of a palm tree branch due to wind as a function of time. In frames (**a**), (**b**) and (**c**) the branch is moving downwards, while in frames (**d**), (**e**) and (**f**) the branch is moving upwards and returning to its original position. The wind speed varies between 4 and 6 m/s.

The movement of a branch of a palm tree due to wind is shown in Fig. 3. The images shown in Fig. 3 were taken on March 2022, and they are for a palm tree in Cairo, Egypt. The measured wind speed at the time of observation varied between 4 m/s and 6 m/s. It can be seen that the branch behaves like a cantilever, i.e. fixed at one end and free on the other end, such that if a force is applied on the free end the cantilever/branch bends. Moreover, the branch is an elastic element, such that if the force acting on the branch, due to wind, is released, the branch returns back to its original position, as shown in Fig. 3. The swinging motion of the branch due to the applied wind force is responsible for the dropping of any deposited dust particles on the leaves. The swinging motion of the leaves does not only assist in cleaning of the leaves but it also prevents the presence of hot spots and burning of the leaves due to a strong solar irradiance. Also, the up and down motion of the leaves due to the applied wind introduces air turbulences that increases the cooling rate of the leaves^34^. It can be concluded that the stronger is the wind the better is the cleaning and cooling of the leaves. On the contrary, the conventional rigid fixture of a PV panel dampens any vibrations due to wind, and will not assist neither in dust removal nor in cooling of the panel.

**Figure 4:**
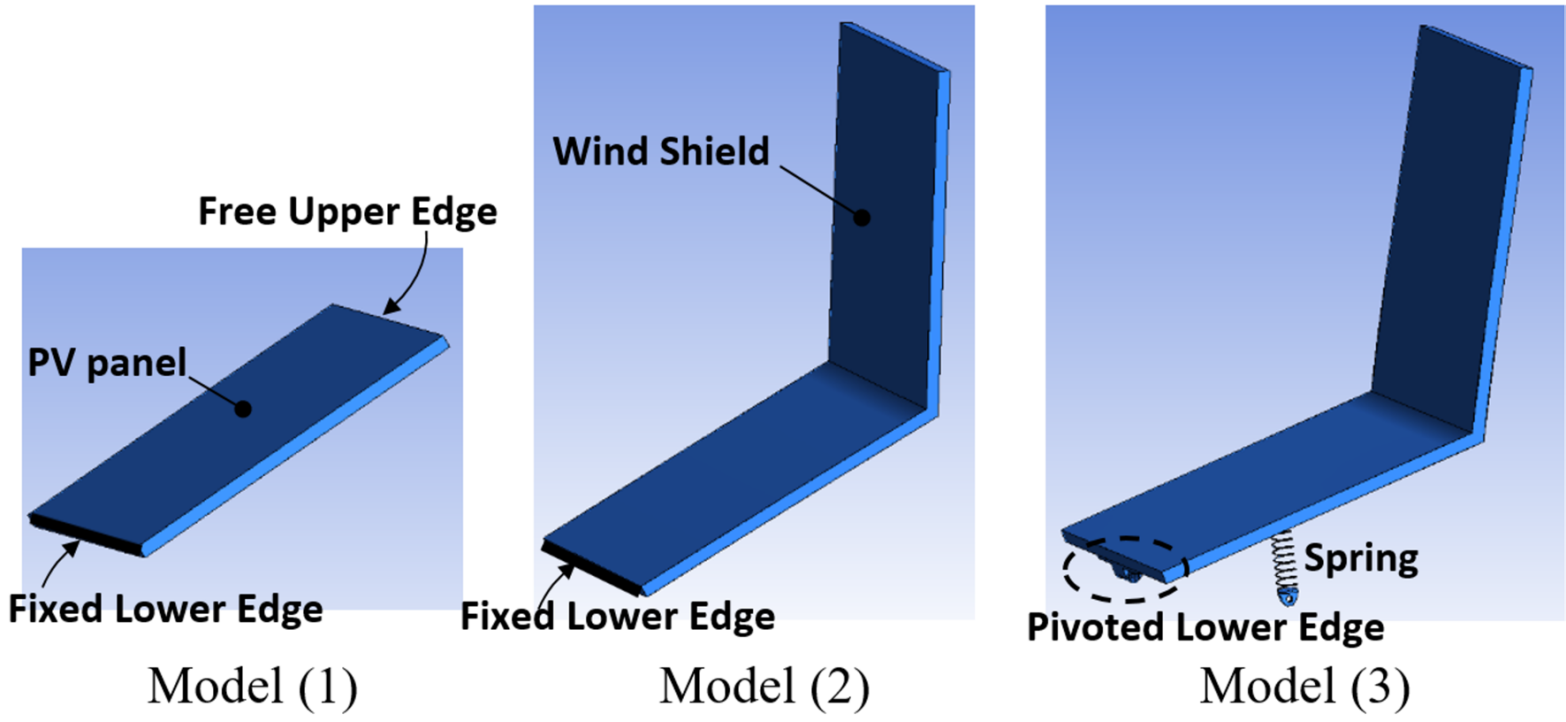
Three different fixation models for the PV panel that have been simulated in Ansys. In model (1) the PV panel is rigidly fixed at its lower edge, in model (2) a vertical wind shield is attached at the upper edge of the PV panel of the 1st model, and in model (3) a spring is installed at the backside of the PV panel of the 2nd model and the PV panel is hinged at its lower edge.

The objective of this study is to design a self-cleaning PV panel that employs the natural vibrations caused by the wind in order to minimize dust accumulation on the panel surface. Three different fixation methods for the PV panel are designed, and the air flow around the panel is simulated using the CFD package Ansys Fluent, while the vibrations and deflections of the panel due to the air flow are simulated using Ansys Mechanical. Ansys System Coupling was employed to coordinate the Ansys Mechanical and Ansys Fluent Solvers in a two way coupling method, to solve this fluid structure interaction (FSI) problem. The different fixation methods are presented in Fig. 4. The first design to be simulated is a PV panel fixed at the lower edge that is simply a cantilever, the second design is similar to the first but a vertical wind shield that has the same dimensions of the panel is attached to it, and the third design is a modification of the second design such that it has a spring located at the back side of the panel and the lower edge of the panel is pivoted, i.e. not fixed, such that the panel can rotate around the lower edge if a force is applied on it. The dimensions of the wind shield is identical to that of the PV panel, which is 0.495 m × 0.43 m. The function of the windshield is to increase the area subjected to wind, in order increase the force acting on the PV panel as well as the attached spring, which subsequently increases the vibration amplitude and oscillations of the panel. Increasing the vibration of the PV panel can assist in decreasing the accumulation of dust over the panel’s surface. All panels where inclined by an angle of 30° above the horizontal. Based on the numerical simulation results, the fixation method of the PV panel that leads to the highest oscillations will be examined experimentally. Experiments are performed to investigate the influence of the proposed new design on dust mitigation of PV panels that operate light posts.

## Numerical methodology

The objective of this numerical study is to determine the influence of wind on the vibration of PV panels. Ansys software was chosen as an accurate and reliable tool to study fluid structure interaction (FSI) on the PV panel. As the wind flows over the PV panel it exerts pressure on the panel, i.e. the structure, that results in moving/deforming the structure, which subsequently affects the fluid flow. This is performed by the two-way system coupling between the fluid and the structure, which involves two data transfers^35^;

1. Pressure from the motion of the air is received by the transient structural analysis system, i.e. Ansys Mechanical, as it solves the structural behavior over time.
2. Displacement data from the motion of the plate is received by the fluid flow analysis system, i.e. Ansys Fluent, as it solves the fluid behavior over time.

Three different fixation models for the PV panel have been designed, as have been shown previously in Fig. 4, and the vibrations due to the applied wind is simulated using Ansys. It has to be mentioned that the PV panel was fixed at its lower edge in case of models 1 and 2, however, the PV panel is pivoted at its lower edge in case of model 3, such that it can revolve around its lower edge.

### Computational domain and boundary conditions

**Figure 5:**
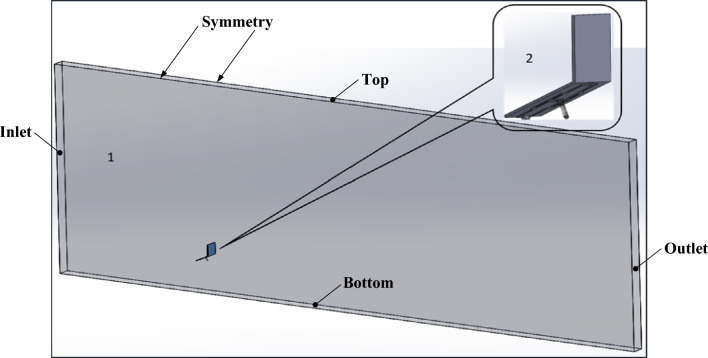
Computational domain, which consists of (1) Fluid domain and (2) the structure, i.e. the PV panel assembly.

The combined geometry which consists of (1) the structure, i.e. the PV panel, mounting base and the wind shield, and (2) the fluid computational domain are shown in Fig. 5. The combined geometry is constructed using the commercial CAD software, Solidworks. The PV panel assembly, i.e. the structure, is imported to the software Transient Structure, while the fluid domain is imported to the Fluent software which are coupled by Ansys workbench. The coupling time step is set to 0.1 s, which is enough to observe the oscillations to a reasonable degree, and the total simulated time is 10 s, which is enough time to observe the plate oscillating few times during the simulation time.

#### Fluid domain

The size of the computational fluid domain shown in Fig. 5 is 21 m × 0.43 m × 9 m, which complies with the guidelines recommended in best practice guidelines^36^. The boundaries of the fluid domain are as follows, (1) the left surface is the velocity inlet, which is named as the “inlet”, (2) the right surface is the pressure outlet, which is named as the “outlet”, (3) the upper and lower surfaces are named as “top” and “bottom”, respectively, the bottom surface which is the ground is modeled as a wall while the symmetry boundary condition is used at the top freestream surface (4) the side surfaces are symmetry surfaces, which are named as “symmetry” and most importantly (5) surface deform, i.e. the surfaces of the computational fluid domain that surround the panel and shield. It has to be mentioned that a 2D simulation is performed, therefore the width of the domain is a slice of the panel and a symmetry boundary condition is applied on the two lateral sides, such that the flow on the rest of the panel is identical to the slice under consideration for the sake of simplicity. However, for a better understanding of the 3D effect, i.e. the lateral flow effect, more simulations will be performed in 3D and the results will be published in the future.

**Figure 6:**
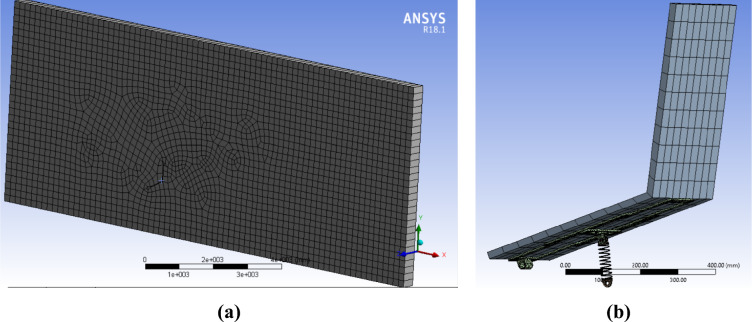
Mesh of (**a**) the fluid domain and (**b**) the structure.

The mesh for the fluid domain is set to be dynamic as the structural deformation causes the surfaces of the computational fluid domain that surround the panel and shield to change their positions with time. The applied mesh is presented in Fig. 6, such that a non-uniform hexahedral mesh was employed in the volume and surfaces of the computational fluid domain, as shown in Fig. 6a. The sweep method was used for domain with single division and with smoothing set to high. The number of nodes and elements for the fluid domain are 10,214 and 4952, respectively. A combination of hexahedral and tetrahedral mesh was automatically generated for the structure as shown in Fig. 6b, which resulted into a total of 6862 elements. The material selected for fluid domain is air with density of 1.184 kg/m^3^ and viscosity of 1.85 × 10^–5^ kg/m^**.**^s, and the inlet air velocity is 4 m/s. The air flow velocity is set to 4 m/s, which is the average wind speed in Cairo, Egypt^37^, in which the experimental work is performed.

#### Structural domain

The dimensions of solar panel are 0.495 m × 0.43 m × 0.018 m, which are the same for the shield. Material assigned to shield and panel is the default structural steel. The contacts are defined in Ansys structure for the different parts of assembly namely; Panel with shield, skeleton, support structure and hinge, mounts and spring. The Young’s moduli of the panel and the spring are taken as 85 MPa and 29 MPa, respectively. The spring used in the numerical simulations is selected based on the weight of the panel and the required vibration limits if the force acting on the spring is increased by ± 10% of the panel’s weight. The force acting on the spring could increase or decrease based on the applied wind direction and magnitude. A + ve force means that the wind force is compressing the spring, while a –ve force indicates that the wind is extending the spring. The spring free length is 14 cm, pitch 1 cm, outer diameter 3 cm, and the wire diameter 3 mm.

The Reynold’s number, Re, for the flow over the PV panel is a function of the distance, x, from the lower edge of the panel and it is governed by^34^,

1 $${\text{Re}} = \frac{\rho Vx}{\mu }$$

where ρ, V and μ are the density, speed and dynamics viscosity of the flow, respectively. Therefore, the maximum Reynold’s number for the flow over the panel is at the upper edge of the panel, and it is calculated based on a flow speed of 4 m/s, density of 1.184 kg/m^3^, viscosity of 1.85 × 10^–5^ kg/m^**.**^s and the characteristic length of the panel is 0.43 m. The maximum Reynold’s number is equal to 1.13 × 10^5^, which is smaller than the critical Reynold’s number, i.e. 5 × 10^5^^34^, for fluid transition from a laminar flow to a turbulent flow over a flat plate. Therefore, the modelling approach used is a 2-way FSI analysis utilizing a Viscous-laminar model, and the assumptions for the transient state simulation comprised a three-dimensional, laminar, and incompressible flow. The CFD code used the Finite Volume Method (FVM) approach and employed the Semi-Implicit Method for Pressure-Linked Equations (SIMPLE) velocity–pressure coupling algorithm with the second order upwind discretization.

### Numerical results

**Figure 7:**
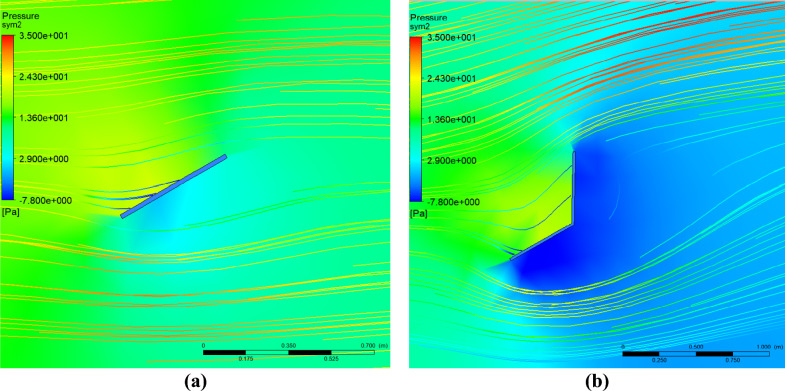
The pressure distribution and the flow field stream lines around the PV panel, in case of fixation models 1 (**a**) and 2 (**b**).

The interaction between a PV panel and the wind blowing over the panel has been numerically simulated using Ansys fluid structure interaction. The pressure distribution and the flow field stream lines around the PV panel at the beginning of simulation, in case of fixation models 1 and 2, are presented in Fig. 7, and it can be seen that there is a pressure build up at the front side of the panel in comparison to the back side. The pressure difference, ΔP, between the front side and the back side in case of model 1 is a about 24.3–(+ 2.9) = 21.4 Pa, while in case of model 2 is about 24.3–(− 7.8) = 32.1 Pa. It can be concluded that the pressure difference between the front and back sides of the PV panel in case of model 2 is larger than that in case of model 1, and that is because of the installed wind shield. Also, the wind shield increases the blockage area of wind, consequently, the force acting on the panel in case of model 2 is larger than that for model 1.

**Figure 8:**
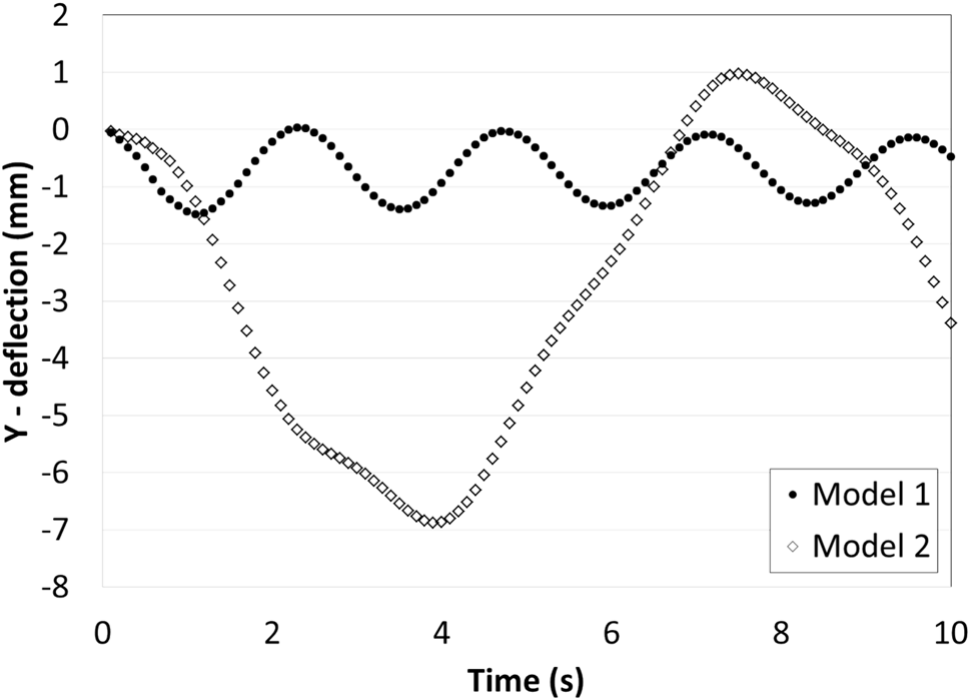
Amplitudes of the vertical deflections of the upper edge of the PV in case of fixation models 1 and 2, as a function of time.

The vertical deflection of the upper edge of the PV panel in case of fixation models 1 and 2 are presented in Fig. 8. It can be seen that the maximum vertical deflection in case of model 2 is much larger than that in case of model 1 because of the added wind shield which increases the wind force acting on the panel. It can be seen from Fig. 8 that the PV panels are oscillating, and that is due to the air flow over the panel. As the air reaches the panel it pushes the panel downwards, and as the air passes the panel, the panel returns back to its original position, due to the elasticity in the panel. The frequency of oscillations in case of panel 1 is larger than that of panel 2 and that is due to the tradeoff between frequency and amplitude of oscillation, since the energy caused by the wind is the same in both cases. So, if the frequency of oscillation increases the amplitude decrease, and if the amplitude of oscillation increases the frequency decreases.

**Figure 9:**
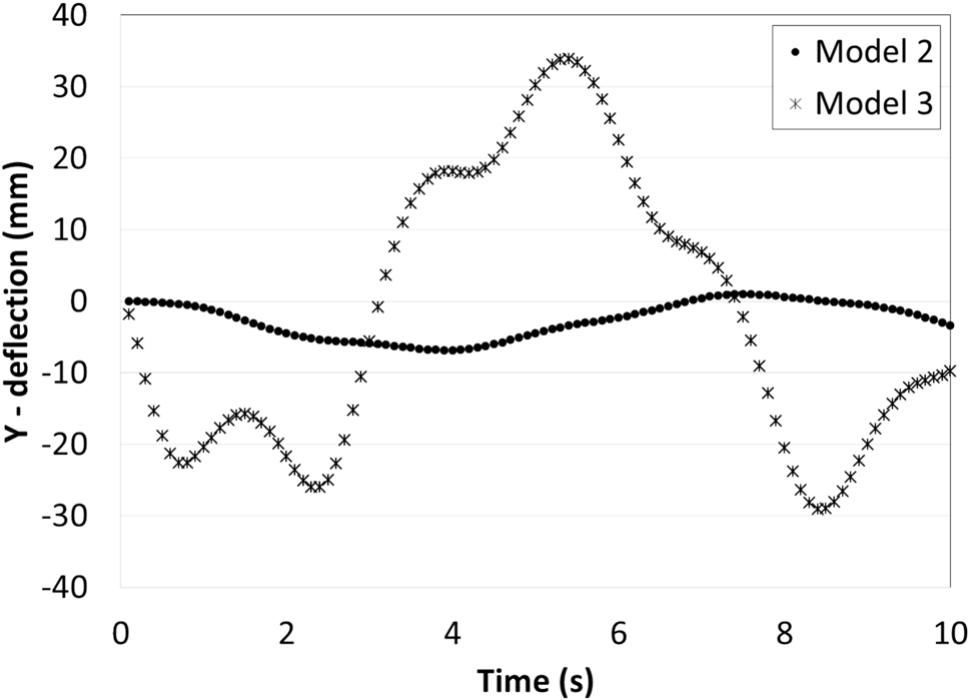
Amplitudes of the vertical deflections of the upper edge of the PV in case of fixation models 2 and 3, as a function of time.

The vertical deflections in models 1 and 2 are below the initial position in most of the simulation time, but in order to minimize the accumulation of dust over the panel’s surface there should be deflections above and below the initial position, also the amplitude of vibration should be as large as possible without breaking the panel. In model 3, the PV panel is hinged at its lower edge in order to ease the movement of the panel, i.e. increase the amplitude of vibration, and a spring is added at the backside of the panel to achieve an overshoot above the initial position. The vertical deflection of the upper edge of the PV panel in case of fixation models 2 and 3 are presented in Fig. 9, and it can be seen that the vertical deflection in case of model 3 is much larger than that in case of model 2, and that is due to flexibility in motion of model 3 compared to model 2. Also, the deflections in model 3 is above and below the initial position, and that is due to the added spring, which stores the spring energy during the downward movement of the panel and releases it when there is no wind force acting on the panel, i.e. when the wind passes the panel. It can be concluded that the fixation model no. 3 is the most suitable design to minimize dust accumulation on the PV panel surface. The influence of the fixation model no. 3 on dust accumulation over the PV panel is examined experimentally and the results are presented in the next section.

## Experimental methodology

Based on the numerical simulations performed, it has been found that a PV panel pivoted at its lower edge, with a vertical wind shield attached at its upper edge and a spring attached at the middle of its backside has the largest vibrations amplitudes due to the applied wind compared to the other designs, consequently, it can be chosen as the most suitable design to minimize dust accumulation over PV panels.

### Experimental setup

**Figure 10:**
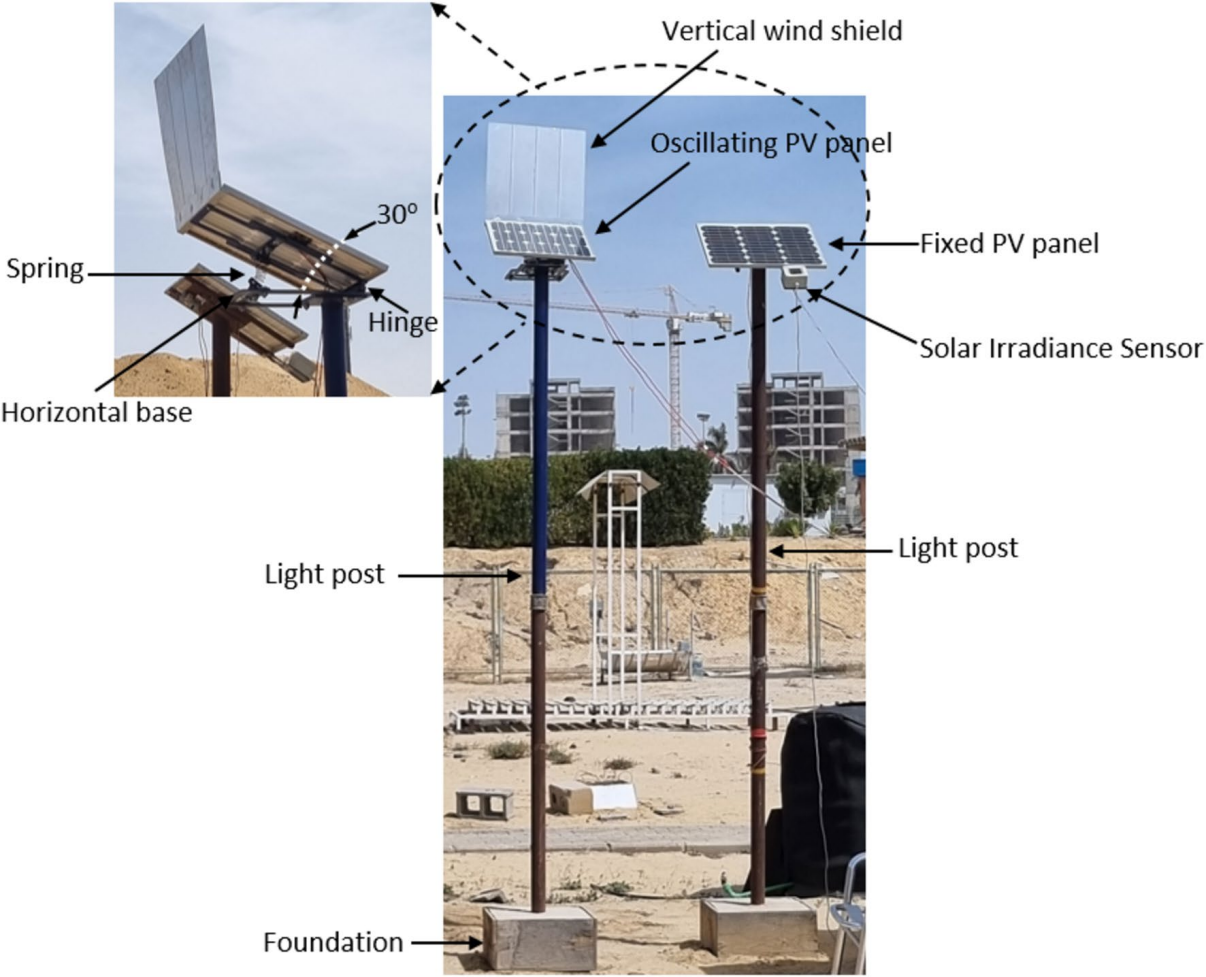
A photo of a PV panel that is firmly fixed to a light post (right) and a photo of the newly fixing method of PV panels operating light post based on oscillations (left). The panels are located in Cairo, Egypt facing the South direction.

Two light posts operated with PV panels are built, as shown in Fig. 10. The first PV panel is rigidly fixed to the light post, i.e. a fixed base, such that it does not vibrate or oscillate due to the applied wind load, while the second panel is flexibly attached to the light post, as have been presented in Fig. 4, such that it can oscillate due to the applied wind load. The chosen design consists of a spring, a hinge and a horizontal base on the light post. The PV panel is connected to the horizontal base via a hinge such that the panel can revolve around the horizontal axis of the hinge. A spring is attached between the panel and the base, such that if the panel is pushed upwards or downwards due to wind it is returned back to its original position. The spring length is adjusted such that the initial angle between the panel and the base is 30°. A vertical wind shield is attached to the panel, as can be seen in Fig. 10, in order to increase the subjected area to wind, consequently, increases the wind force acting on the panel.

### Experimental procedure

The PV panels were cleaned manually by water before the start of the experiments, and no further cleaning was performed during the entire period of the experiments. Both PV panels were coated by the nano-particles coating, which is manufactured by Sketch Co., Ltd., Tokyo, Japan^38^, which is an antistatic-hydrophilic nano-coating for glass and solar panels. The experiments were conducted for 6 consecutive weeks, starting from the 3^rd^ of April till the 15^th^ of May, 2022, in Cairo, Egypt. The conventionally fixed panel was taken as a reference panel for comparison, i.e. a PV panel without any modifications. The efficiencies of the PV panels were measured for two days per week. The following parameters including; irradiance, temperature, voltage and current at the maximum power point were measured in each experiment each 5 min for 2 h starting from 8:00 a.m. till 10:00 a.m. And based on these parameters, the efficiency of the PV panels was calculated using the following equation,

2 $$\eta = \frac{{V_{mpp} \times I_{mpp} }}{{A_{c} \times G}}$$

where V_mpp_ and I_mpp_ are the voltage and current at the maximum power point, G is the solar irradiance and A_c_ is the area of the PV panel. The percentage drop in efficiency of the PV panel is calculated using the following equation,

3 $$\% \Delta \eta = \frac{{\eta_{c} \times \eta_{avg} }}{{\eta_{c} }} \times 100$$

where η_c_ is the efficiency of the PV panel under clean conditions, i.e. on the 1^st^ day of experimentation, and η_avg_ is the average efficiency of the PV panel during any day of measurements. The maintenance limit for cleaning, was taken as 10%, i.e. when the drop in efficiency of the PV panel due to dust accumulation exceeds the 10%, cleaning of the panels should be done.

### Experimental results

**Figure 11:**
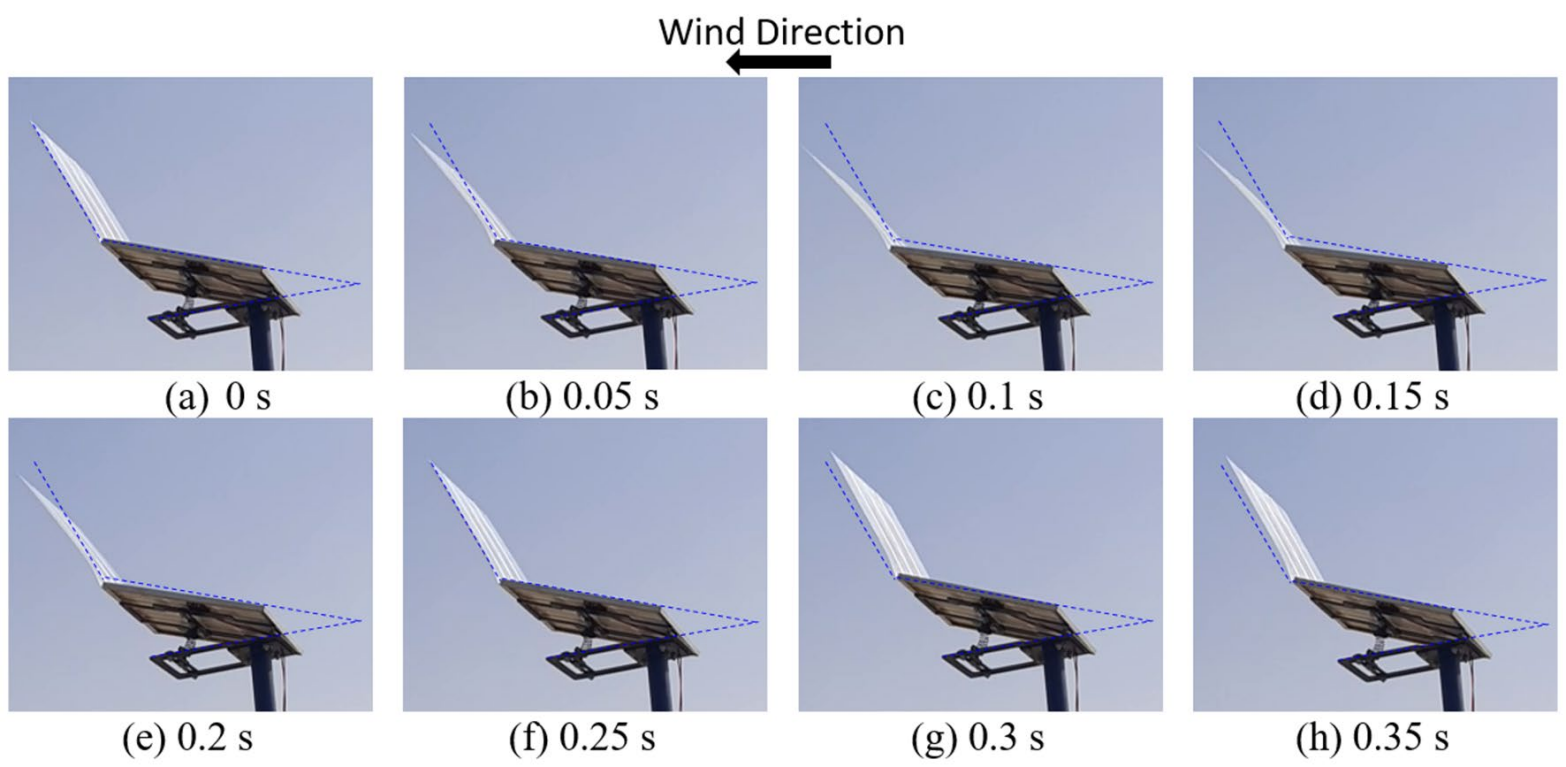
Oscillation of the PV panel due to wind as a function of time. In frames (**a**), (**b**), (**c**) and (**d**) the panel is pushed forward in the direction of wind, while in frames (**e**), (**f**), (**g**) and (**h**) the panel is moving backward to its original position by the effect of the attached spring. The wind speed varies between 4 and 6 m/s.

The oscillation of the PV panel due to winds is shown in Fig. 11. It can be seen that the PV panel is pushed forward by the wind and then it returns back to its original position by the effect of the attached spring. This oscillation of the PV panel can assist in dust mitigation over the panel together with the antistatic-hydrophilic coatings.

**Figure 12:**
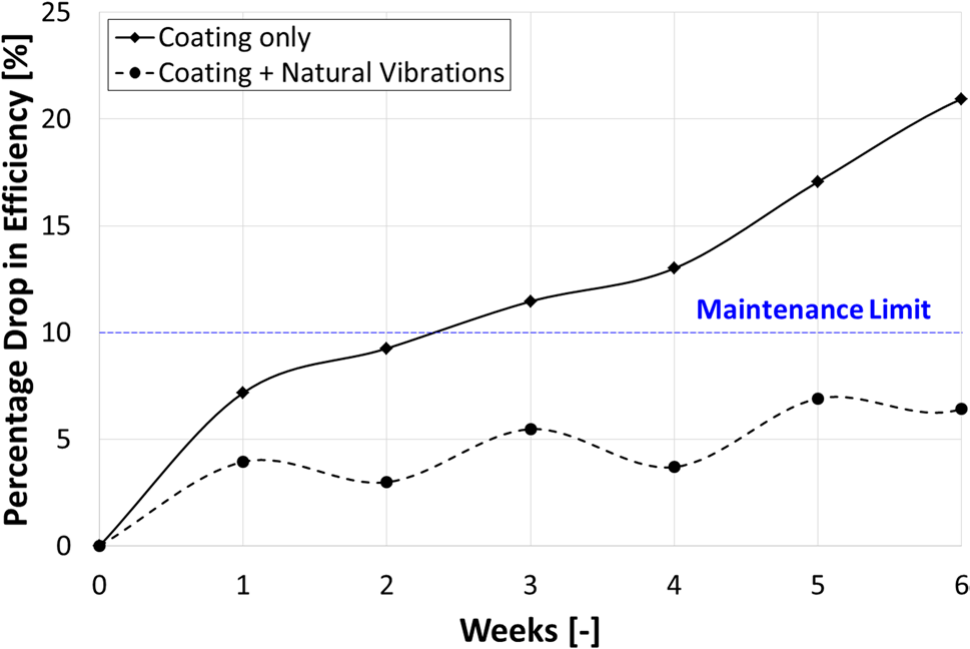
Percentage drop in the efficiency of the PV panel as a function of time in case of the panel is (**a**) only coated and (**b**) coated plus vibrated due to the applied winds. The measurements were taken in Cairo, Egypt in April 2022.

The Percentage drop in the efficiency of the PV panel as a function of time in case of the panel is (a) only coated and (b) coated plus naturally vibrated due to the applied winds is shown in Fig. 12. The measurements were taken in Cairo, Egypt in April 2022. It can be seen that percentage drop in efficiency of the panel, which is rigidly fixed to the light post, is increasing with time, and it has exceeded the maintenance limit^32^, i.e. 10% drop in efficiency, in the 3^rd^ week of operation. The maintenance limit is defined as the limit at which soiling of the panel becomes substantial and cleaning is a must. However, the percentage drop in efficiency of the flexible PV panel is oscillating below the maintenance limit, and it did not exceed the maintenance limit till the end of the performed experiment, i.e. after 6 weeks of operation. The oscillations in the efficiency of the PV panel is due to the variation in vibration of the PV panel due to the existing winds, which are very strong in some weeks that causes strong vibration of the panel, and consequently immense cleaning of the panel, while in other weeks the winds are weak which can marginally vibrate the panel. The percentage drop in efficiency of the fixed PV panel has exceeded the 21% after 6 weeks of operation, while the flexibly fixed PV has only reached 6.5%, which indicates the importance of vibrations on dust accumulation on the panel’s surface. It can be concluded that the oscillating motion of the PV panel is the main reason for dust mitigation on the panel, and in order to minimize dust accumulation over a PV panel operating a light post, the panel should be fixed on a flexible base that allows the panel to vibrate as the wind blows over it causing the dust to fall off the surface.

## Conclusions

The objective of this research is to develop a fixation method for PV panels, such that the panel can vibrate as the wind blows in order to minimize dust accumulation. Three different fixation methods for the PV panel are designed, and the air flow around the panel is simulated using the CFD package, Ansys Fluent, while the vibrations and deflections of the panel due to the air flow are simulated using Ansys Mechanical. The first design simulated is a PV panel that is fixed at its lower edge and free at the upper edge, which is simply a cantilever, and the second design is similar to the first except that a vertical wind shield is attached to the panel at its upper edge. The third design is a modification of the second design such that it has a spring located at the back side of the panel and the lower edge of the panel is pivoted, i.e. not fixed, such that the panel can rotate around the lower edge if a force is applied on it. The PV panel in the first and second design is rigidly fixed, while it is free to oscillate in the third design, i.e. it is flexibly fixed. Experiments have been performed to examine the influence of installing a PV panel on a flexible and a rigid base on dust accumulation over the panel. Based on the performed research it can be concluded that;

1. The PV panel that is flexibly fixed has the largest vibration amplitude due to the applied wind compared to the PV panel that is rigidly fixed.
2. The drop in efficiency of the flexibly fixed PV panel did not exceed the maintenance limit, i.e. 10%, during the course of experiment, which was 6 weeks, while the drop in efficiency of a rigidly fixed PV panel has reached 21% after 6 weeks of operation.
3. Installing a PV panel on a flexible base, such that the panel can vibrate as the wind blows over it, is a passive solution for minimizing dust accumulation over the panel.
4. Further research should be done to determine the influence of vibrating the panel on preventing hot spots as well as cooling of the panel.

## Data Availability

All data generated or analysed during this study are included in this published article.
